# Cell polarity signaling in the plasticity of cancer cell invasiveness

**DOI:** 10.18632/oncotarget.7214

**Published:** 2016-02-08

**Authors:** Aneta Gandalovičová, Tomáš Vomastek, Daniel Rosel, Jan Brábek

**Affiliations:** ^1^ Department of Cell Biology, Charles University in Prague, Viničná, Prague, Czech Republic; ^2^ Institute of Microbiology, Academy of Sciences of The Czech Republic, Videňská, Prague, Czech Republic

**Keywords:** polarity, invasion, plasticity, EMT, AMT

## Abstract

Apico-basal polarity is typical of cells present in differentiated epithelium while front-rear polarity develops in motile cells. In cancer development, the transition from epithelial to migratory polarity may be seen as the hallmark of cancer progression to an invasive and metastatic disease. Despite the morphological and functional dissimilarity, both epithelial and migratory polarity are controlled by a common set of polarity complexes Par, Scribble and Crumbs, phosphoinositides, and small Rho GTPases Rac, Rho and Cdc42. In epithelial tissues, their mutual interplay ensures apico-basal and planar cell polarity. Accordingly, altered functions of these polarity determinants lead to disrupted cell-cell adhesions, cytoskeleton rearrangements and overall loss of epithelial homeostasis. Polarity proteins are further engaged in diverse interactions that promote the establishment of front-rear polarity, and they help cancer cells to adopt different invasion modes. Invading cancer cells can employ either the collective, mesenchymal or amoeboid invasion modes or actively switch between them and gain intermediate phenotypes. Elucidation of the role of polarity proteins during these invasion modes and the associated transitions is a necessary step towards understanding the complex problem of metastasis. In this review we summarize the current knowledge of the role of cell polarity signaling in the plasticity of cancer cell invasiveness.

## INTRODUCTION

Cell movement is an important process in every multi-cellular organism, central to morphogenesis especially in organisms lacking cell walls. In metazoans, it is required not only during development but also in adult organisms, where it is essential during wound healing, immune responses, maintaining tissue homeostasis, tissue renewal and integrity. These processes are carefully controlled and when derailed, the excess of cell migration can cause severe pathological states such as disintegration of tissues or fibrosis. An overt example of pathological consequences of deregulated cell migration is dissemination of cancer cells from the primary tumor and formation of secondary tumors, metastases, in distant organs and tissues [1–3].

**Figure 1:**
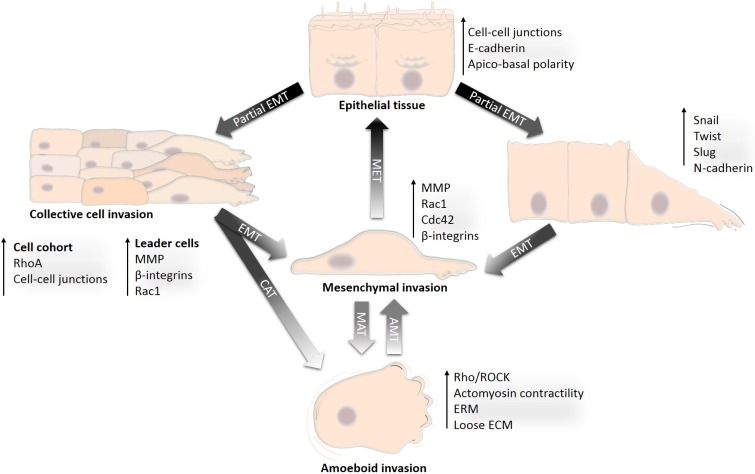
Development of diverse invasion modes from differentiated epithelium Transition from the differentiated non-motile epithelium to a motile and invasive state is a gradual process during which cancer cells acquire diverse invasion modes. The non-motile state is represented by differentiated epithelial cells. Acquisition of an invasive phenotype is a result of a multistep process of cancer-associated EMT. Incomplete or partial EMT can induce collective migration in which cells can retain cell-cell adhesions and migrate collectively in a coordinated manner as sheets or cell clusters. Cells that undergo complete EMT often lose contact with the cell cohort or detach from the epithelial sheet, establish front-rear migratory polarity and migrate individually in the mesenchymal mode. Mesenchymally migrating cells may re-differentiate by mesenchymal-epithelial transition (MET) and re-establish an epithelium. Alternatively, by losing dependency on ECM and by increasing actomyosin contractility, mesenchymal cells can undergo mesenchymal-amoeboid transition (MAT) and invade in the amoeboid mode. The amoeboid phenotype could also be achieved by an increase in Rho activity in collectively migrating cells, which then undergo the collective-amoeboid transition (CAT); however, this is less frequent than MAT. The amoeboid and mesenchymal modes of invasion are often inter-convertible, and amoeboid cells can also revert to mesenchymal mode by amoeboid-mesenchymal transition (AMT).

The ability of cancer cells to move through the extracellular matrix and invade the surrounding tissue requires the transition of differentiated cells, organized in a non-invasive tissue, to cells of a migratory and invasive phenotype. This transition is almost invariantly accompanied by changes in the cell shape, as exemplified in epithelial cells that are the origin of the majority of solid cancers. In epithelial cells, the loss of epithelial cuboidal or columnar shape and gain of mesenchymal-like elongated morphology is typical of invasive cancer cells (Figure 1). Such a profound change in cellular shape relies on the loss of apical-basal polarity and establishment of front-rear polarity in migrating cells. Thus, establishing either apical-basal or front-rear axis is crucial for the cell non-invasive or invasive behavior, respectively [4–6]. In this way, the transition from epithelial to migratory polarity may be seen as the hallmark of cancer progression to an invasive and metastatic disease [7] (Figure 1).

How cancer cells induce the loss of the apical-basal polarity and acquire the migratory phenotype is still an open, unresolved question. Evidence suggests that the tumorigenic factors in cooperation with the tissue microenvironment awaken a programmatic switch by which cancer cells suppress epithelial features and gain the mesenchymal and invasive characteristics. This phenotypical switch is often considered to be a subtype of the epithelial-mesenchymal transition (EMT), a developmentally encoded process associated with embryonic development or physiological injury [8, 9]. During tumor progression, cancer associated EMT represses the function of polarity and cell-cell adhesion complexes and, on the other hand, induces expression of mesenchymal and pro-migratory genes (reviewed in [9, 10]). However, not all components that govern the apical-basal polarity and cell-cell cohesion are repressed during EMT. Rather, these components are re-utilized in migrating cells. In fact, they form novel signaling pathways nonexistent in differentiated cells and re-route the upstream signaling towards a migratory and invasive outcome.

It is tempting to view the transition from a static to a migratory and invasive phenotype as a one-step linear switch. In reality, the migratory phenotype is the result of a gradual process encompassing several intermediate steps, which can be stable or transient, and mutually exclusive or interconvertible (Figure 1). As a result, cancer cells can utilize different modes of migration ranging from single cell invasion, invasion in cell cohorts to invasion in multicellular sheets [11]. This flexibility and the ability to adapt to extracellular conditions is the reason why metastatic cancer is such a problematic disease and the limited success of therapeutic anti-metastatic intervention reflects it.

In this review we focus on the mechanisms and processes underlying the plasticity of cell invasion that were documented in metazoans, as a rule vertebrates, unless stated otherwise. The ability of cancer cells to hijack the components of apical-basal polarity and re-utilize them to promote cell migration and invasion emerges as a common theme of cancer cell migration and invasion. To do that, epithelial polarity components form atypical signaling connections, which ultimately converge in order to regulate Rho GTPases and promote cell motility.

## EPITHELIAL POLARITY COMPONENTS AND COMPLEXES

### The architecture of differentiated epithelial cells

**Figure 2:**
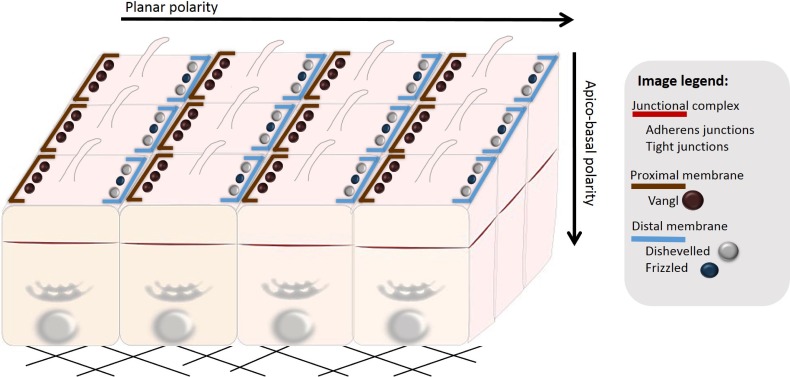
Polarization of epithelial cells Epithelial cells organized in a multicellular epithelium are polarized both in the vertical (apico-basal polarity) and horizontal direction (planar cell polarity). Apico-basal polarity depends on spatial distribution of polarity complexes (see Figure 3). Planar cell polarity (PCP) is established by asymmetrical localization of PCP proteins along the proximal and distal membranes, which ensures polarization of cells in the direction orthogonal to apico-basal polarity. PCP coordinates cellular behavior of cells present in a multicellular epithelium (here exemplified by uniformly aligned cilia).

Differentiated epithelial cells present in the cohesive cell layer exhibit apico-basal polarity (vertical direction; Figure 2) and planar cell polarity (horizontal direction; Figure 2), which define the cell shape, its position and function within a tissue. The apico-basal polarity is determined by specific localization of different cell adhesion complexes, namely tight junctions (TJs) and adherens junctions (AJs) [12, 13]. TJs are found between cells at their apical side and they consist of transmembrane proteins occludin, claudin, tricellulin and JAMs (junctional adhesion molecules). TJ proteins are engaged in homo- and heterophilic cell-cell interactions at the extracellular side and form the seal between cells. On the cytoplasmic side they are associated with scaffolding proteins, such as ZO-1 (zonula occludens 1) that couple TJs to the perijunctional cytoskeleton. Adherens junctions (AJs) are located just below TJs and mediate cell-cell cohesion through the homophilic intercellular interaction mediated mainly by E-cadherin. On the cytoplasmic side E-cadherin associates with a group of cytoplasmic catenin proteins (p120-catenin, β-catenin and α-catenin) that connect AJs to cytoskeletal structures [12, 13]. The AJs and TJs, collectively termed the junctional complex, divide the cell into apical and basolateral regions. On the basal side, epithelial cells are attached to the extracellular matrix (ECM) proteins of the basal lamina through transmembrane proteins of the integrin family. The cellular organization with integrin adhesions at the basal side, cell-cell adhesions at the lateral membranes, and the junctional complex separating apical and lateral membranes is typical of differentiated epithelial cells [12, 14] (Figure 2).

Cell-cell and cell-ECM adhesions are connected to cytoskeletal filaments, and to actin in particular. Multiple points of extracellular adhesions linked to the cytoskeleton reinforce the epithelial cell shape and function. Consequently, the differentiated epithelial tissue is manifested as cuboidal or columnar cells organized in multicellular cohesive tissue with limited paracellular permeability [12, 14]. The important aspect of the tight association of cells through the junctional and adhesion complexes is that it limits the migratory and invasive potential of polarized cells. Concordantly, remodeling of the intercellular adhesions and loss of the apical-basal polarity has been recognized as an important step in the acquisition of a motile and invasive phenotype [15].

In addition to apico-basal polarity, planar cell polarity (PCP) is established within epithelial tissue by coordinated alignment of epithelial cells in response to global directional cues. The establishment of PCP results in the asymmetrical distribution of signaling components along the axis orthogonal to the apical-basal polarity axis [16] (Figure 2). Although it is unclear whether the loss of PCP predisposes the tissue for cancer development, several pieces of evidence suggest that the deregulation of PCP complexes has tumorigenic potential [17].

### Apico-basal cell polarity complexes define the epithelial cell shape

**Figure 3:**
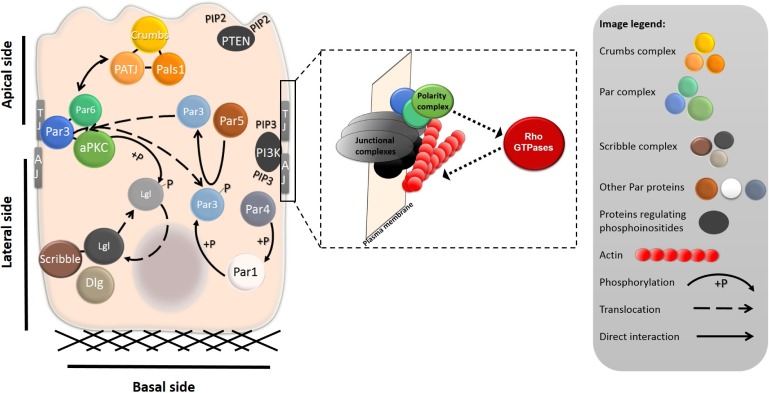
Intracellular localization of polarity complexes in a differentiated epithelial cell The shape of a differentiated epithelial cell is governed by the cell-ECM and cell-cell adhesions at the basal and lateral sides of the cell, respectively. At the basal side, cells are attached to the ECM through the adhesion plaques primarily composed of integrins and integrin-associated proteins (not shown). The tight junctions (TJ) and adherens junctions (AJ) complexes separate apical and basolateral membranes and promote establishment of the apico-basal polarity. The localization and mutual interactions between polarity complexes and proteins are important for the establishment and maintenance of apico-basal polarity. The Crumbs complex, composed of proteins Crumbs, PATJ and Pals1, localizes to the apical membrane. The Par complex, which includes aPKC and Par3 and Par6, is localized laterally, where it interacts with TJ proteins. Scribble, Lgl and Dlg form the Scribble complex, which is basolateral. Mutual interactions, most notably phosphorylation, regulate the segregation of the proteins to the apical or basolateral side. The enlarged part shows the functional interdependence of junctional complexes, polarity complexes, and Rho GTPases. The polarity complexes (depicted in green-blue) localize to junctional complexes (grey-black) or membranes of epithelial cells. Junctional complexes or diverse upstream signaling molecules can activate the polarity complexes, which, in turn, regulate the activity of Rho GTPases. Rho GTPases through their effectors control actin polymerization (actin is depicted in red).

Key factors responsible for establishment of the apico-basal polarity are three evolutionarily conserved protein complexes Par (Partitioning defective), Scribble and Crumbs. As they promote the epithelial differentiated phenotype, they are considered to be tumor suppressors [18–20]; however, recently they have also been shown to contribute to the regulation of malignant progression [21]. The signaling pathways of Par, Scribble, Crumbs and phosphoinositides are mutually regulated and interconnected with small Rho GTPases signaling through recruiting specific guanine nucleotide exchange factors (GEFs) and GTPase activating proteins (GAPs) for RhoGTPases. Ultimately, they spatially control the actin cytoskeleton to promote the establishment and maintenance of cell-cell junctions and polarized phenotype (Figure 3).

**Figure 4:**
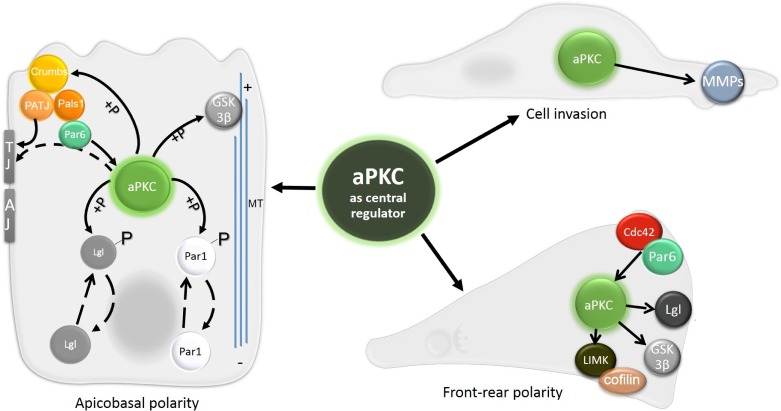
The central role of aPKC in apico-basal polarity, front-rear polarity and cell invasion Signaling events regulated by aPKC are central for establishing both apico-basal and front-rear polarity. aPKC phosphorylates Crumbs to enhance its apical localization, and proteins Lgl and Par1 to promote their basolateral localization. GSK3-β, involved in microtubule stabilization, is also a target of aPKC. Targeting aPKC to the site of TJ through interaction with Par6-Pals1-PATJ is also shown. The establishment of front-rear polarity relies on spatial distribution of Cdc42 and Par6, which can activate aPKC at the leading edge. There it phosphorylates LIMK, GSK3-β and Lgl. aPKC also directly promotes cell invasion by upregulating and activating MMPs.

Among the polarity complexes, Par has the widest range of functions. First identified in *Caenorhabditis elegans*, the Par complex is located at the apical side within the region of tight junctions (Figure 3, Table 1) and consists of Par3, Par6 and aPKC (atypical protein kinase C, aPKCι or aPKCζ isoforms in human). Generally, the Par complex promotes formation and maintenance of the tight junctions and apical membrane [22]. Par3 and Par6 are the PDZ domain containing proteins that mediate protein-protein interaction and associate with several proteins including tight junctions proteins and aPKC [23, 24]. Upstream of the Par complex are Rho GTPases Rac1 and Cdc42 that associate with the Par complex and activate aPKC [25, 26]. aPKC activation is the central event in the regulation of apico-basal polarity as aPKC phosphorylates several polarity substrates including Crumbs, Lgl and GSK3β (glycogen synthase kinase-3β) (Figure 4). Phosphorylation of Crumbs and Lgl promotes their correct intracellular localization (see below for details). GSK3β phosphorylation controls the capture and stabilization of microtubules [27] and cell-cell contacts maturation [28]. In addition to microtubules, the Par complex controls actin dynamics by regulating Rac1 activity. For example, the recruitment of Tiam1 (T lymphoma invasion and metastasis), a GEF for Rac1, is important for epithelial polarization as it promotes perijunctional actin polymerization and tight junctions formation [29]. These data implicate the existence of a positive loop that reinforces junctional complexes formation (Figure 3).

**Table 1:** Localization of polarity proteins and Rho GTPases in epithelial cells and in migrating and invading cells

Polarity protein		Epithelial cells	Collective cell migration		Individual cell migration	
			leader cells	cell cohort	amoeboid	Mesenchymal
Par complex	Par6	tight junctions region	leading edge	retains epithelial distribution	midbody (leukocytes)	leading edge
	Par3					
	aPKC					
Other Par proteins	Par1	basolateral	?	cell-cell contacts	?	?
	Par4	cytoplasm	?	?	?	leading edge
Crumbs complex	Crumbs	apical membrane	leading edge	retains epithelial distribution	?	leading edge
	PATJ					
	Pals1					
Scribble complex	Scribble	basolateral membrane	leading edge	retains epithelial distribution	uropod (leukocytes)	leading edge
	Lgl					
	Dlg					
Phosphoinositides	PTEN	apical membrane	rear	retains epithelial distribution	rear	rear
	PI3K	adherens junctions	leading edge		leading edge	leading edge
RhoGTPases	RhoA	actomyosin ring	rear	actomyosin ring; protein activity downregulated	cell cortex	rear
	Rac	cytoplasm	leading edge	cytoplasm	leading edge, protein activity downregulated	leading edge, protein activity upregulated
	Cdc42					
Planar cell polarity proteins	Vangl1	?	?	?	?	leading edge
	Vangl2	cell-cell boundaries	?	retains epithelial distribution	rear	?

The table summarizes the localization of the most important players of cellular polarity and their changes during acquisition of the motile and invasive phenotype. In several cases, the localization of polarity proteins is not known (indicated by a question mark).

The proper function of the Par complex requires its exclusion from the basolateral region and association with the apical cortex. The exclusion is attained by coordinated action of a 14-3-3 protein Par5 and kinases Par1 and Par4. These proteins are not part of the Par complex, but are essential for polarity regulation. To exclude the Par complex from the basolateral domain kinase Par1 phosphorylates Par3, and Par5 mediates shuttling of phosphorylated Par3 by direct binding (described further below, Figure 3). Par4 (also known as LKB1), is likely upstream of Par1 as it has been shown to phosphorylate and activate Par1 kinase both in *Drosophila* and in mammals [30, 31] (Figure 3). Intriguingly, Par4 is considered a tumor suppressor often lost or mutated in human cancers (reviewed in [32–34]).

Along with Par, the Crumbs complex also localizes to the apical side, particularly to the apical membrane (Figure 3, Table 1). It consists of the transmembrane protein Crumbs and two associated proteins - Pals1 (protein associated with Lin seven 1) and PATJ (Pals1 associated tight junction protein). PATJ is a scaffold protein with a PDZ domain. Its partners include tight junction proteins ZO-3 and claudin [35]. It is therefore not surprising that PATJ has been shown to promote formation of tight junctions [36, 37]. Crumbs complex also interacts with the Par complex and, at least in *Drosophila,* Crumbs promotes Par complex apical localization (described further below, reviewed in [38]). Moreover, the Crumbs complex directly contributes to spatially restricted activation of Rho GTPases as it recruits Rich, a GAP for Cdc42, to the TJs region [39]. In addition, Crumbs components recruit Rho GEFs Syx and p114RhoGEF that increase Rho activity (reviewed in [38]).

Unlike Par and Crumbs complexes, the Scribble polarity complex is localized basolaterally (Figure 3, Table 1). The core of the Scribble complex is formed by conserved proteins Scribble, Dlg (Disc large) and Lgl (Lethal giant larvae). Scribble and Dlg proteins contain PDZ domains, similarly to Par3, Par6, Crumbs or PATJ. Through its PDZ domain Scribble associates with vimentin. Interestingly, PDZ domains of Dlg bind several products of proto-oncogenes such as APC (adenomatous polyposis coli), PTEN (Phosphatase and tensin homolog) and β-catenin [40–42]. The interaction with β-catenin appears to target Scribble to the E-cadherin-β-catenin complex along the lateral membrane, where it further stabilizes cell adhesions [43]. Functionally, Scribble is engaged in an antagonistic relationship with the Par complex (see below, Figure 3). It also interacts with a Rac and Cdc42 GEF, βPIX, indicating that it controls actin remodeling (reviewed in [38]).

### Planar cell polarity complexes

Planar cell polarity proteins cooperate to generate polarity in the direction orthogonal to the apico-basal axis. PCP coordinate cellular processes polarized across the tissue plane such as oriented cell division and cilia function [16]. PCP proteins are part of the non-canonical Wnt (β-catenin independent) signaling pathway. The most important PCP proteins include receptor proteins Van Gogh (Vang; also known as Strabismus) and Frizzled (Fz), and the adaptor protein Dishevelled (Dsh). The most common ligand of mammalian PCP signaling is Wnt5 [44]. PCP proteins are initially localized in the cytoplasm. During the establishment of PCP they translocate to the membrane, where they asymmetrically distribute between proximal and distal membranes. For example, upon polarization in cochlear hair cells, Vangl2 localizes uniformly to the proximal cell-cell boundaries [45] (Figure 2, Table 1).

The deregulation of PCP components can contribute to the loss of epithelial structures, an important step towards collective cell migration and invasion [46]. Furthermore, interactions with both apico-basal polarity protein complexes and Rho GTPases have been documented [47] (see chapter 5).

### Mutual interactions and asymmetric localization of polarity signaling components

The polarity complexes Par, Scribble and Crumbs engage in antagonistic and cooperative interactions that reinforce their polarized localization (Figure 3). In an antagonistic manner, the Par3/Par6/aPKC complex controls the basolateral localization of the Scribble complex component Lgl. When Lgl translocates to the apical side it is phosphorylated by aPKC resulting in re-localization to the lateral region [48]. Similarly, the phosphorylation of Par1 by aPKC excludes Par1 from the apical domain. The laterally localized Par1 further promotes apico-basal asymmetry by phosphorylating Par3, which is consequently excluded from the basal region. This mechanism involves 14-3-3 protein Par5. Par5 binds phosphorylated Par3 and serves as a shuttle from the lateral membrane to the cytoplasm, where upon dephosphorylation Par3 dissociates from Par5 [49] (Figure 3).

In contrast to Scribble, the Crumbs complex acts cooperatively with the Par complex to regulate Par localization to TJs. This cooperative effect is mediated by a direct interaction between Par6 and Pals1 [37]. It was further shown that Pals1 affects aPKC localization [36]. The proposed mechanism is that through binding of PATJ, which directly binds TJ proteins, Pals1 can recruit Par6 and thus aPKC to the TJ region (Figure 4).

Conversely, Crumbs is directly phosphorylated by aPKC and this phosphorylation is indispensable for the correct apical localization of Crumbs and PATJ in *Drosophila* epithelial cells [50]. Altogether, aPKC seems to be the key mediator in establishing the apico-basal polarity. It not only keeps Lgl at the basal side, but also maintains the localization of the Crumbs complex at the apical region (Figure 4).

Planar cell polarity complexes are generaly considered to act indepedently of apico-basal complexes in epithelial cells, however; there is a crosstalk between them during developmental processes and ciliogenesis. Par1 can e.g. phosphorylate and promote Dishevelled translocation to the cell cortex during *Xenopus* development [51], and the Par complex is required for the formation of cilia [52]. Notably, PCP complex interacts with the Scribble complex to postulate the front-rear polarity of migrating cells (discussed in chapter 5). Interestingly, it has been hypothesized that the Par complex and PCP components are mutually antagonistic although the molecular mechanisms remain unknown [53].

Taken together, asymmetric localization of the polarity complexes provides subcellular cues for the polarized organization of the cytoskeleton. The major regulators of the cytoskeleton, Rho GTPases, can function both upstream and downstream of the polarity complexes (Figure 3). Their activity must be spatiotemporally balanced as either hyper-activation or inhibition of the particular Rho GTPase can compromise epithelial polarity.

## LOSS OF EPITHELIAL POLARITY AND GAIN OF CELLULAR INVASIVENESS

### Cellular reprogramming by EMT

The destabilization of junctional complexes and actin cytoskeleton, remodeling cell-matrix adhesions, and overall loss of epithelial phenotype accompanied with the expression of a pro-invasive set of genes is characteristic of the progression toward an invasive and malignant phenotype of most carcinomas. EMT utilized by cancer cells has been attributed as a programmatic switch leading to the acquisition of the mesenchymal mode and the development of invasive cancer (Figure 1). The transforming growth factor-β (TGFβ) signaling pathway plays a prominent role in inducing EMT; however, receptor tyrosine kinases, Wnt, Hedgehog or Notch signaling pathways can synergize with TGFβ signaling or induce EMT independently of TGFβ. Importantly, the activation of multiple proto-oncogenes such as Ras, PI3 kinase/AKT and Src can also initiate EMT and increase the invasive and metastatic potential of cancer cells [54].

The phenotypical changes associated with EMT depend on both non-transcriptional and gene expression reprogramming. In the classical view the activation of transcription factors such as Snail/Slug, Twist or ZEB is the main trigger of EMT [55–57]. These transcription factors suppress expression of multiple epithelial genes and simultaneously induce the expression of genes typical of migratory mesenchymal cells. One of the hallmarks of EMT is the “cadherin switch”, which is characterized by the exchange of E-cadherin for N-cadherin. The cadherin switch results in reduced intercellular cohesion and is associated with poor prognosis in carcinomas [58]. Another hallmark of EMT is the intermediate filaments switch, which suppresses the epithelial keratins and induces the expression of vimentin.

### EMT alters the function of polarity complexes to induce loss of epithelial polarity

The loss of epithelial polarity during EMT indicates that the activation of the EMT program affects the function of polarity complexes and consequently the integrity of cell-cell junctions. Because EMT represses the transcription of several epithelial genes it is reasonable to expect that polarity complexes could also be affected on gene expression level. Indeed, EMT-associated transcription factors Snail and ZEB1 have been shown to affect expression of the polarity complexes. Snail represses Crumb3 at the gene expression level, resulting in the disappearance of Crumbs, but also the Par complex from cell-cell junctions [59]. In addition, Snail can suppress Lgl expression by binding to its promoter region. Lgl repression then induces invasive behavior, which can be reversed upon re-expression of Lgl [60]. Notably, the tumor suppressor Par4/LKB1 can suppress Snail1 levels and thus inhibit the metastatic behavior of cells [61]. Another EMT promoting transcription factor ZEB1 represses Crumbs, PATJ and Lgl along with several TJ and AJ proteins by directly binding their promoter regions [55]. The polarity protein Scribble can also affect expression of epithelial proteins, as the loss of E-cadherin during EMT could be induced by Scribble knockdown [43].

Other studies link the polarity proteins with the TGFβ pathway, which can impair the function of polarity proteins both at the transcriptional and non-transcriptional level. On transcriptional level, TGFβ signaling represses Par3 expression and disrupts the Par complex [62]. On non-transcriptional level, TGFβ receptor type II co-localizes with Par6 at tight junctions and phosphorylates Par6 [63]. Phosphorylated Par6 binds to the ubiquitin ligase Smurf and targets it to TJs. Here, Smurf locally degrades RhoA leading to the loss of tight junctions and the induction of EMT [63]. The TGFβ and Par6 signaling axis appears to be developmentally encoded as they also participate in axon formation in the developing brain [64]. Interestingly, aPKC has recently been shown to co-localize with TGFβ receptors. Similarly to TGFβ, aPKC also phosphorylates Par6 boosting the ability of Par6 to target RhoA for degradation [65]. Furthermore, the aPKC-Par6 complex interacts with another growth factor receptor - ErbB2. ErbB2 does not phosphorylate Par6, however, by recruiting the aPKC-Par6 complex, ErbB2 disrupts the apico-basal polarity in non-tumorigenic mammary epithelium cells and induces the formation of abnormal multi-acinar structures [66].

### Epithelial-mesenchymal transition as origin of cell invasion plasticity

Permanent gene expression reprogramming is typical of complete EMT and reinforces the stable mesenchymal migratory phenotype of cells. However, depending on the cell type and cellular context, cells can exist in several intermediate metastable phenotypic states described as incomplete EMT, partial EMT, or EMT-like phenotype. The molecular mechanisms that predispose cells for complete or partial EMT subtypes remain poorly understood but are clearly cell type dependent. For example, in some cell types ERK can induce complete EMT with repressed E-cadherin [67]. However, in other cell types ERK activation does not affect E-cadherin expression but rather induces its removal from the cell membrane and internalization [68, 69]. It thus appears that EMT covers the broad spectrum of phenotypically and functionally different states ranging from multicellular epithelium to autonomously migrating cells. We suggest that these functional and phenotypical differences, namely the variations in intercellular cohesion and expression of pro-migratory genes, are the origin of cell invasion plasticity, manifested as the different modes of cell migration.

**Figure 5:**
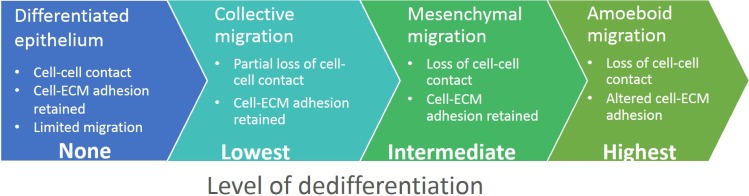
Cell invasion modes in the context of epithelial dedifferentiation We hypothesize that the plasticity of cancer cell invasion originates from gradual dedifferentiation of epithelial cells. In this model, well-developed epithelium retains both cell-cell and cell-ECM adhesions and represents the differentiated state. In the collectively migrating cell cohort, leader cells partially dedifferentiate and gain some mesenchymal characteristics, but they also retain some epithelial features such as cell-cell cohesion. The completion of EMT results in the loss of epithelial features and gain of a cell autonomous mesenchymal-like mode of invasion. These cells lose cell-cell contacts but actively form adhesions with the ECM. Finally, cells utilizing the amoeboid invasion mode lose both cell-cell and cell-ECM adhesions, resembling the most dedifferentiated state.

Provided that the epithelial phenotype is characteristic of differentiated cells, then the loss of epithelial features can be seen as a dedifferentiation process (Figure 5). If we perceive EMT as a group of reversible and mutually interconvertible steps, we find that the cancer cell invasion modes each resemble a certain stage of EMT. Simplified, these steps are: loosening of cellular junctions; loss of all cell-cell junctions and the gain of a pro-invasive phenotype; altered cell-ECM adhesion and increased migratory potential. We propose a model that recognizes the cancer cell invasion modes as gradual dedifferentiation accompanied by the loss of epithelial characteristics. From this perspective, collective cell migration responds to the least dedifferentiated with amoeboid migration being most dedifferentiated (Figure 5).

An additional source of heterogeneity in tumors is provided by the reversibility of EMT. Cells migrating individually in a mesenchymal manner can undergo a process opposite to EMT, during which they lose motility, settle, retain cellular junctions and differentiate to form multicellular epithelial structures (Figure 1). This mesenchymal to epithelial transition (MET) has been proposed as the primary mechanism of establishing a secondary tumor in tissues [70]. It has been documented e.g. in colon carcinomas, where both primary tumors and secondary metastasis form well-differentiated epithelial structures while disseminating cells display the characteristics of mesenchymal cells [71].

## MODES OF CELL INVASION

### Collective cell migration and invasion

Collectively migrating and invading cells display heterogeneous appearance as they migrate in the form of strands, sheets or cell clusters that can greatly differ in cell numbers, ranging from a few cells to large masses of cells. The invading cell cohort may either stay connected to the primary tumor or detach and migrate independently and even enter a blood or lymphatic vessel [72]. Within the cell group, cells maintain intercellular contact mediated by adhesion molecules such as cadherins [73]. Notably, the contacts are strong enough to keep the cell mass together when migrating through heterogeneous ECM [74].

While the majority of the cells resemble tightly coupled epithelial cells, the cells in the front of the migrating sheet frequently display mesenchymal characteristics or various epithelial-mesenchymal intermediate phenotypes [75]. These “leader”, “guiding” or “pioneer” cells weaken or loose cell-cell contact. Similar to mesenchymal cells, leader cells have dynamic actin cytoskeleton with actin rich protrusions. Furthermore, these cells display higher levels of membrane proteases capable of degrading the ECM and thus they generate migrating tracks for the cell cohort, often referred to as “following” cells [76]. The mechanism of collective cell migration is still not fully understood, however, it is evident that the Rho GTPases play principal role. The activation of Rho GTPases differs in “leader” cells and the cell cohort [77] (Table 1). Rac, Phosphoinositide 3-kinase (PI3K) and β1-integrins are preferentially localized to the front of leader cells [78] where Rac activation and actin polymerization generate the protrusive forces.

Rho activity during collective migration is carefully balanced at a certain level, which differs in leading and following cells. The shift from equilibrium may lead to both enhanced and disrupted migration. The excess of Rho activity increases actomyosin contractility resulting in the disruption of cell-cell junctions and single cell migration [79], whereas the inhibition of Rho in epithelial cells during wound healing induces the formation of leader cells with mesenchymal features promoting migration of the cell sheet [80]. Moreover, in finger-like protrusions formed during wound healing, the highest Rho activity has been detected at the sides of the fingers where it prevents Rac mediated protrusions [81].

Overall, collective migration seems to follow the rule of contact inhibition of locomotion [82], which describes the fact that cells tend to form new protrusions towards sites lacking cell-cell contact. In the context of collective cell migration, this imposes the role of leader cells expanding forward rather than pushing against the cell cohort. Correspondingly, the migrating leader cells impose pulling forces on the following cell cohort through intercellular adhesions to induce collective movement. However, evidence suggests that the followers are not a simple cargo, but actively participate in the migration and invasion process. The following cells may promote forward movement by increasing cell number by proliferation [83]. Moreover, in a polarized epithelial sheet migrating into the wound, following cells are capable of forming lamellipodial protrusions termed “cryptic lamellipodia”. Cryptic lamellipodia spread beneath the neighboring cells and convey signals throughout the cell mass [84] demonstrating that collective cell migration requires the cooperation of both leader and following cells. How the cell cohort responds to extracellular cues and ECM topology has been recently summarized elsewhere [85].

Additionally, the migrating cohort forms cell contacts with surrounding “accessory” cells. In collective cancer cell invasion, cancer-associated fibroblasts function as accessory cells. They can take up the role of leader cells and further promote invasion by remodeling the ECM [86].

Collective migration was recently proposed to be the prevalent mechanism for the detachment of cancer cells from the tumor mass [87]. By reconstructing 3D images of the tumor surroundings it was shown that most cells maintain contact with each other when invading the ECM.

### Individual cell invasion - the mesenchymal mode

Cells that undergo complete EMT adopt the mesenchymal mode of invasion (Figure 1). They invade individually, without the need of any cell-cell contact, however, they retain cell adhesion to the ECM. Typically, these cells are elongated and utilize surface bound proteases to partially degrade the ECM making space to move forward.

Simultaneous degradation of the ECM and formation of adhesive structures that generate traction forces underlies the mesenchymal mode of invasion. The adhesive structures, focal complexes and focal adhesions, are multimolecular assemblies of both structural (e.g. integrins, talin, vinculin, paxillin) and signaling (e.g. focal adhesion kinase, Src) proteins that provide a mechanical link between intracellular actin bundles and the ECM [88]. The dynamic formation and disassembly of cell-ECM adhesions is important for the generation of traction forces, ECM remodeling and cell rear retraction [89, 90]. During migration, the focal complexes formed at the cell front either disassemble or mature into focal adhesions. The disassembly of adhesion complexes is mediated by the ERK kinase, which localizes to focal adhesions [91, 92]. On the other hand, Rho mediated actomyosin contractility induces stabilization and maturation of focal adhesions [93], and also their sliding at the cell rear [94].

Besides focal adhesions, mesenchymally migrating cells also form special adhesion structures called invadopodia and podosomes (sometimes termed collectively invadosomes or podosome-type adhesions, PTA). Both podosomes and invadopodia are formed at the site of cell-ECM contacts and are built of a core rich in F-actin and actin regulatory proteins such as Arp 2/3, cortactin or WASP. The core is surrounded by a ring composed of integrins and adapter proteins vinculin and paxilin that, similarly to focal adhesions, links ECM to the actin cytoskeleton. Importantly, mature invadosomes contain proteolytic enzymes, which corresponds to their role as ECM-degrading structures [95, 96].

The proteolytic activity is provided by enzymes capable of degrading components of the ECM: MMPs (matrix metalloproteases), ADAMs (a disintegrin and metalloproteinase), cathepsin proteinases, and serine proteinases such as urokinase-type plasminogen activator. Matrix metalloproteases are expressed as inactive pro-enzymes and to become activated proteolytical processing is necessary. Subsequently, MMPs are recruited to the integrin-ECM binding site of invadopodia [97] and degrade the adjacent ECM making space for the cell's forward movement [98–100]. The ECM degradation can be observed as a tube-like matrix defect that trails the invasion pathway [99, 101]. Since MMPs can facilitate invasion it cannot come as a surprise that the up-regulation of specific MMPs in tumors has been confirmed, [102, 103]. Both focal adhesion turnover and degradation of the ECM are limiting factors regarding the invasion speed which is approximately 0.1 - 1 μm/min [104].

### Small GTPases signaling and actin dynamics in mesenchymal cell polarization, migration and invasiveness

**Figure 6:**
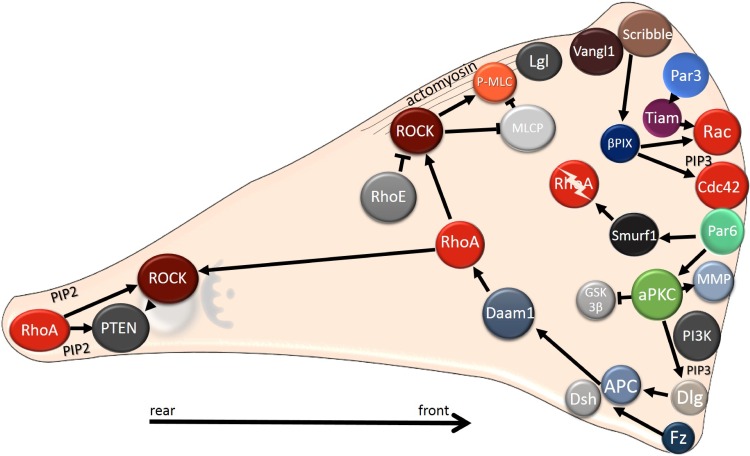
Intracellular localization of polarity proteins during mesenchymal migration Polarity proteins localize to the leading edge, where they regulate Rho GTPases. At the leading edge, Cdc42 and Rac are activated, whereas the RhoA protein level is reduced as RhoA is degraded by Smurf. The Rho/ROCK pathway is stabilized at the cell rear by a positive feedback loop comprising lipid phosphatase PTEN and PIP2. In mesenchymal cells Par and Crumbs complexes co-localize with PIP3 (and PI3K) to the cell front, in contrast to epithelial cells, where the Par and Crumbs complexes co-distribute with PIP2 to the apical region. The Scribble complex, which is found basolaterally in epithelial cells, localizes to the leading edge, where it regulates Rho GTPases. Note that in mesenchymally migrating cells Rho could also be activated at the cell front (not shown, see text for details).

The whole process of mesenchymal invasion requires the establishment of cell polarity characterized by distinct spatial distribution of Rho GTPases, adhesion molecules, and second messengers (Figure 6, Table 1). These signaling molecules then cooperate in defining the spindle or conical cell shape with lamellipodial protrusions induced at the cell front and limited on the cell sides and rear [105]. In most polarized cells, the nucleus is located at the cell rear and the microtubule organizing center (MTOC) is positioned toward the leading edge, forming the nuclear-centrosomal axis aligned with the direction of migration. Both microtubules and actin cytoskeleton are specifically arranged along the nuclear-centrosomal axis and reinforce cell polarization and thus directional migration [106, 107]. The establishment of the polarized profile is largely controlled by Cdc42. In an integrin-dependent manner, Cdc42 induces nuclear and MTOC re-localization and microtubule stabilization at the cell front [106, 108]. However, the role of Rho GTPases in cell polarization appears to be cell type or context specific, as the nuclear movement and establishment of the nuclear-centrosomal axis in some cell types could also be regulated by Rho signaling [109–111].

Cell migration of polarized cells is driven by dynamic actin reorganization. In this way, dendritic actin polymerization induces the formation of protrusions specifically at the cell front, where they are stabilized by the attachment to ECM through integrin-mediated adhesions. Protrusions are initiated by Rac1 and Cdc42, which cooperate through the WASP/SCAR/WAVE family of proteins that activate Arp2/3 mediated actin polymerization. Another protein that drives actin polymerization and directional protrusivity is the actin severing protein cofilin. Cofilin enhances actin turnover by severing actin filaments and creating new barbed ends, and its activation is sufficient to induce the formation of new protrusions [112]. The activity of cofilin is inhibited by several mechanisms including phosphorylation by LIM and TES kinases, binding to PIP2, or cortactin [113]. Active cofilin is found at the tip of the leading edge or in invadopodia and, intriguingly, it has an important role in promoting directional migration [114, 115]. In agreement, constitutively active cofilin promotes metastasis of prostate tumors and its increased expression was detected in human metastatic tumors [116].

Rac1 signaling, that is central in mesenchymal migration, is primarily activated in response to extracellular stimuli. In the case of adhesion signaling that is mediated by the integrin receptor family, Rac1 is activated in a focal adhesion kinase dependent manner. Its activation is further reinforced by a positive feedback loop. The integrins from Rac-induced focal complexes activate PI3K, which localizes to the leading edge and produces PIP3 [117]. This second messenger is known to bind a Rac1 specific GEF, Tiam1 (T lymphoma invasion and metastasis) [118]. Moreover, Tiam1 is able to directly bind a subunit of Arp2/3 and thus localize to the actin branching point. There it recruits and activates Rac1, which subsequently activates Arp2/3. In this way, a positive feedback loop regulating the dynamics of actin protrusions is established [119]. Another adhesion signaling pathway leading to Rac activation includes DOCK3, a Rac1 specific GEF, which can interact with CAS/Crk. CAS belongs to a family of adaptor proteins in focal adhesions that form a complex with Crk, which binds DOCK3, thus recruiting and activating Rac at the site of focal adhesions [120]. In addition, suppression of Rho activity at the cell front contributes to Rac activation as Rac and Rho are mutually antagonistic [121, 122]. In parallel with adhesion signaling, the activation of the Rho GTPases and tumor cell invasion is directly affected by chemical signals present in the tumor microenvironment. In particular, chemokines and growth factors produced by stromal cells and the activation of their cognate receptors is critical for cancer cell migration and invasion (reviewed in [123, 124]).

In contrast to the cell front, where PIP3 accumulates along with GTPases Rac1 and Cdc42, the cell rear has higher concentrations of PTEN and its product PIP2 [125, 126] (Figure 6, Table 1). The main GTPase at the rear is Rho and its effector kinase ROCK whose activation induces the assembly of thick contractile stress fibers anchored at adhesions. Rho/ROCK activate actomyosin contraction by promoting phosphorylation of myosin light chain (MLC) [127, 128]. Rho/ROCK mediated assembly and contraction of stress fibers is involved ECM remodeling and cell polarization [129–131]. Rho/ROCK signaling is also important for rear retraction that depends on FAK (focal adhesion kinase) mediated activation of PDZ-RhoGEF [94]. Thus, Rho induces contraction to keep the cell rear in contact with its front, while Rac initiates protrusions that tend to stretch forward. The role of Cdc42 is mainly to maintain cell polarity, i.e. directional migration.

Although the prominent role of RhoA is at the rear, spatiotemporal studies showed that RhoA also participates in the formation of the leading edge [132–134]. In fact, its activation precedes Rac1 and Cdc42 activation that serves to promote the protrusions formation [135]. Additionally, RhoA also contributes to membrane ruffling [136] and actin retrograde flow [134]. These studies challenge the common view that Rho activity is low at the cell front and high at the cell rear. Indeed, there is evidence emerging that the asymmetrical activation of Rho downstream effectors ROCK and mDia (Diaphanous-related formin) play a role in cell polarization [137]. In line with these results is the finding showing that RhoC activity is controlled by RhoGEFs and RhoGAPs at the leading edge and the site of invadopodia, where it controls phosphorylation of the actin severing protein cofilin [138]. It has been proposed that precise spatiotemporal regulation of RhoC at lamellipodia and invadopodia plays a central role in directional migration and invasion, respectively [139].

### Individual cell invasion- the amoeboid mode

Amoeboid invasiveness is thought to be independent of matrix degradation and largely independent of cell-ECM adhesion. In agreement, cells naturally utilizing the amoeboid mode of invasion display low expression of β1-integrins [140]. In the absence of strong cell-ECM attachment and ECM degradation the movement of amoeboid cells is enabled by contractions of the cortical actomyosin network leading to membrane blebbing [141]. Bleb formation is driven by the cell cortex, i.e. the cortical actomyosin network and associated proteins, which separates from the cell membrane by hydrostatic pressure of the cytoplasm. These events predispose amoeboid cells to move by a bleb-driven mechanism during which the invading cell squeezes through the holes in the surrounding 3D network of ECM filaments. Cells utilize the bleb to move forward either by forming weak, transient adhesions or by contracting the rear and pushing forward [142]. The high cell deformability leads to one magnitude higher invasion velocities compared to mesenchymal invasion [143]. In fact, amoeboid cancer cells disseminating from a primary tumor have been shown to migrate at the speed of 15 μm/min [144].

Cells migrating in an amoeboid manner have reduced dependency on both cell-cell and cell-ECM adhesion. Hence, amoeboid migration corresponds to the least differentiated state (Figure 5). In agreement with this hypothesis, the gain of an amoeboid phenotype was associated with stem-like features of melanoma cells [145]. Moreover, it was shown that the expression of pluripotency genes Nanog and Oct4 in melanoma cells induced expression of amoeboid-specific genes [146].

### Small GTPase signaling in amoeboid invasiveness

**Figure 7:**
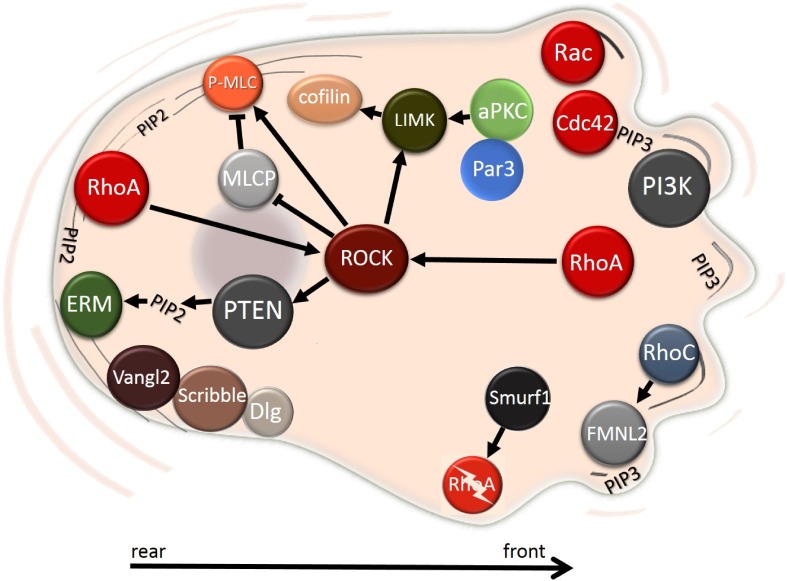
Intracellular localization of polarity proteins during amoeboid migration The Rho/ROCK pathway is the most prominent signaling hub during the amoeboid migration. PI3K localizes to the front, while PTEN remains at the rear, where it activates ERM proteins by producing PIP2. The Par complex regulates actin assembly through LIMK. Planar cell polarity Vangl2 localizes to the rear of the cell along with proteins from the Scribble complex. See text for details.

The most prominent signaling pathway in amoeboid migration is Rho/ROCK (Figure 7). Upon activation by Rho, ROCK enhances contractile forces by increasing the phosphorylation of MLC2. Mechanistically, ROCK activates myosin light chain kinase (MLCK) [128], which subsequently phosphorylates MLC2. In addition, ROCK inhibits the activity of myosin light chain phosphatase (MLCP) that dephosphorylates MLC2 [127]. Apart from ROCK-induced inhibition, MLCP activity is also reduced by phosphorylation by zipper-interacting protein kinase (ZIPK) [147] or by myotonic dystrophy kinase-related Cdc42-binding kinase (MRCK), which is activated by Cdc42 [148]. In result, both phosphorylation of MLCK and MLCP leads to increased levels of phospho-MLC2, which activates the myosin II motor activity. Notably, ROCK contributes to the localization of MLC into actin bundles at the cell cortex. These bundles orientate perpendicularly to the direction of movement to generate force needed for movement [149]. Altogether, Rho/ROCK manages the overall and local ratio between phosphorylated MLC2 and un-phosphorylated MLC2, which determines the level of cell contractility [150]. Nevertheless, other regulators of the actin network also contribute to the final outcome. For example, RhoC and its target formin FMNL2 (Formin-like protein 2) were found to promote amoeboid cell motility by inducing actin assembly [151].

The actomyosin contractility is opposed by Cdc42 and Rac1 and their effector p21-activated protein kinase 1 (PAK1). PAK1 directly inhibits MLCK by phosphorylation [152], indicating that Cdc42 and Rac1 dampen the amoeboid mode of invasion. However, in some cells, Cdc42 and its upstream activator DOCK10 can promote amoeboid motility, as Cdc42 activates PAK2 and consequently actomyosin contraction [153].

In amoeboid cells, the migratory polarity is less evident than in mesenchymal cells. Nevertheless, the polarized spatial distribution of PTEN, PI3K and their products is the main characteristic of amoeboid cells (Figure 7, Table 1). In *Dictyostelium discoideum,* PI3K localizes to the cell front without any need of a chemoattractant [154] while PTEN co-localizes with myosin II at the rear [155]. In mammalian cells PI3K is also found at the cell front [156], however, PTEN does not clearly localize to the rear, but rather it is distributed throughout the cytosol [126, 157]. Actived PTEN binds to the membrane and produces PIP2, which promotes directional amoeboid movement by enhancing the stiffness of the cell cortex. PIP2 regulates localization of the ERM (ezrin-radixin-moesin) proteins that crosslink actin filaments with the plasma membrane. This has been described in melanoma cells where PIP2 and ezrin co-localize to the plasma membrane of the retracting rear. Along with phosphorylated MLC they form a rigid structure that was named ERULS (ezrin rich uropod-like structure). ERULS significantly reduces bleb formation at the cell rear, which increases directional movement [158]. Interestingly, it was suggested that blebs and lamellipodia are polarized structures and share a common mechanism that determines the site of their formation. This was demonstrated by repeated Rac1 activation and deactivation, which lead to switching between blebs and lamellipodia. Intriguingly, they both formed at the same site [159].

The localization of proteins of the polarity complexes during amoeboid migration has been described in migrating leukocytes. Dlg and Scribble were shown to localize to the uropod, i.e. the trailing end, while Par3 remained in the cell body [160] (Figure 7). Overall, the Par complex was shown to be important for directional migration in leukocytes as its disruption resulted in impaired enrichment of F-actin at the cell rear [161].

## PLASTICITY OF INVASION - TRANSITIONS BETWEEN INVASION MODES

Cells migrating in one invasion mode are often able to employ another mode (Figure 1), and this plasticity of cell invasion and migration appears to be an important reason why metastatic cancer is such a problematic disease to intervene with. The observations that mesenchymal and amoeboid invasion modes are driven by increased Rac and RhoA signaling, respectively, raised the hypothesis that strengthening either pathway may lead to a change in the invasion mode and contribute to plasticity of migration and invasion (reviewed in [121, 162]). In most cases Rac and RhoA function in an antagonistic manner, [122] and, in agreement, modeling the activity of Rho GTPases in conjunction with the migration mode revealed that preferential activation of either Rho or Rac could be a decisive factor for the establishment of a phenotypically stable invasion mode [163].

It is important to take into consideration that the invasion mode is only to some extent determined by the cell origin and type and that most cell lines have subpopulations of both invasion modes. Several studies have shown that the migration mode is largely dictated by the tumor microenvironment, particularly by the rigidity and composition of the ECM. For example, mesenchymal migration is preferentially used in stiffer matrices, whereas more loose matrices allow amoeboid motility [164–166]. This is true not only for cancer cells as the migration mode of human macrophages is also dependent on the ECM architecture [167]. Importantly, blocking essential components of either invasion mode can lead to the switch to the second mode, which can represent an escape mechanism for tumor cells during the treatment of invasive cancers.

It must also be emphasized that even though the amoeboid and mesenchymal modes of migration seem to be viewed as distinct, opposite invasion strategies, individually migrating cells are able to take advantage of both mesenchymal and amoeboid characteristics [168].

### The mesenchymal-amoeboid transition

Individually migrating cells can effectively change their phenotype to switch the modes of invasion, undergoing transition from amoeboid to mesenchymal (AMT) or from mesenchymal to amoeboid (MAT) phenotype (Figure 1). The induction of MAT in cancer cells often occurs after the weakening of cell-ECM adhesions by reducing the concentration of fibers in the ECM, by activating the Rho signaling pathway [143], or by blocking a critical component of invasion such as MMPs [99]. In addition, the Rho/ROCK pathway can suppress the mesenchymal mode of invasion by activating ARHGAP22, a GAP for Rac, thus lowering the activity of Rac. Accordingly, the silencing of ARHGAP22 by siRNA induced a mesenchymal cell phenotype [120]. A reverse effect was observed after silencing of Rac activators DOCK3 and NEDD9. Knockdown of DOCK3 and NEDD9 lead to increased MLC2 phosphorylation, which is typical of amoeboid motility [120]. Another pathway identified in the regulation of MAT is RhoA degradation mediated by E3 ubiquitin ligase Smurf1. By targeting RhoA for proteosomal degradation, Smurf1 plays an important role in cancer cell invasion (Figures 6 and 7). Silencing Smurf1 in mesenchymal colon cancer cells resulted in MAT and elevated migration levels [169].

Given the central role of Rho in amoeboid invasion, the Rho effectors of the formin family may be expected to be important players in the transition between amoeboid and mesenchymal invasion. Formin mDia1 was found to be essential for RhoA/ROCK-dependent blebbing [170], while family member mDia2 (also known as diaphanous-related formin 3 or DIAPH3) paradoxically supports formation of invadopodia [171], typical of mesenchymal cells. Correspondingly, mDia2 expression suppressed the amoeboid phenotype while its loss was associated with a rounded cell shape, membrane blebbing and also elevated levels of metastasis [172]. An additional regulatory mechanism of cell migration downstream of Rho GTPases involves activation of LIM kinases LIMK1 and LIMK2. ROCK activates both LIMK1 [173] and LIMK2 [174], whereas Rac1 preferentially activates LIMK1 [175]. In mesenchymally migrating fibrosarcoma HT1080 cells, overexpression or activation of LIMK1 led to MAT, as cells adopted the rounded amoeboid phenotype. In these cells, LIMK1 activation was induced by MMP inhibitors and was dependent on the Rho-ROCK signaling pathway. However, depletion of LIMK1 suppressed both amoeboid and mesenchymal invasion. Therefore, LIMK plays a role in amoeboid migration, where it contributes to actomyosin contraction, but also in mesenchymal migration, where it presumably influences formation of lamellipodia [176].

### Amoeboid-mesenchymal transition

Unlike MAT, the reversal process of AMT is poorly documented. As mentioned above, the mesenchymal phenotype can be induced by silencing ARHGAP22, which leads to an increase in Rac activity [120]. Recently, AMT was induced in cells naturally utilizing the amoeboid invasion after inhibiting the Rho pathway by silencing the glycoprotein NG2 [140]. Another study identified DOCK10 and Cdc42 to be closely related to the amoeboid phenotype by influencing MLC phosphorylation through MRCK. Silencing DOCK10, a GEF specific for Cdc42 shifted the invasion mode from the rounded amoeboid to the elongated mesenchymal phenotype [153].

### Collective-amoeboid transition

Collectively migrating and invading cells can also undergo transition to autonomously migrating cells. They can either gain a mesenchymal phenotype by undergoing complete EMT, or switch to amoeboid movement in a process of collective to amoeboid transition (CAT) (Figure 1). During CAT cells dissociate from the migrating cohort by loosening cell-cell and integrin-ECM adhesion and gain amoeboid characteristics. Although CAT is the least common, it has been observed in melanoma cells upon inhibition of β1 integrins [177].

Similarly to MAT, it appears that CAT also requires elevated Rho activity. A recent study showed that the cleavage of EphA2, a receptor often upregulated in invasive cancers, increases RhoA activity which results in collective-to-amoeboid transition in breast carcinoma cells [178]. Interestingly, EphA2 is cleaved by matrix metalloprotease MMP-1 and co-localizes with it to the cell membrane [178]. Elevated RhoA activity has also been implicated in CAT of collectively migrating human bronchial epithelial cells. In these cells depletion of myosin-IXA, a protein containing Rho-GAP activity, resulted in disrupted cell-cell contacts and cell scattering. The individually migrating cells displayed blebbing typical of amoeboid invasion [79]. The role of myosin-IXA in maintaining epithelial architecture requires its interaction with ZO-1. This interaction recruits myosin-IXA to the site of cell-cell contact, where it locally inhibits RhoA via its RhoGAP domain [79]. These results suggest that Rho activation at cell-cell junctions is required for junctional disassembly and for the establishment of the amoeboid invasion mode. Counterintuitively, Rho activity can also lead to the assembly of tight junctions, as shown for p114RhoGEF mediated Rho activation [179]. However, during amoeboid and collective migration p114RhoGEF promotes invasion by stimulating MLC phosphorylation [180]. Overall, these results suggest that collective and amoeboid migration both require enhanced actomyosin contractility. On the other hand, leading cells are dependent on cell-ECM adhesions and form protrusions in the direction of movement [181], a feature typical of mesenchymal movement. It is therefore evident that collective invasion takes advantage of the traits of both single cell migration modes.

## THE FUNCTION OF APICO-BASAL AND PLANAR CELL POLARITY COMPONENTS IN CELL INVASION

The physiological functions of polarity proteins are almost invariantly altered in cancer cells so as to assist tumor progression. Their deregulation can be either in terms of quantity or in terms of localization, both leading to aberrant downstream signaling. Par polarity complex proteins Par3 and aPKC are examples of aberrantly expressed proteins in tumors. Par3 is often deleted in human cancers being considered tumor suppressor [182], while aPKCι is overexpressed, which led to the establishment of aPKCι as an oncogene [183]. On the other hand, Scribble is commonly found to be delocalized in cancer cells [184, 185]. The altered expression or mutations of polarity proteins with implications for oncogenesis have been reported elsewhere [15, 186–188] and here we thus focus on the role of polarity proteins in cell migration and invasion (summarized in Table 1).

### Apico-basal polarity complexes in single cell migration

#### The interaction of polarity complexes with Rho GTPases

Cdc42 and the Par complex emerge as key factors in the establishment of migratory cell polarity, a step prerequisite for cell migration. Activated Cdc42 binds Par6 to promote the activity of aPKC [23] (Figure 4). Activated aPKC phosphorylates and inhibits GSK-3β and, under these conditions, the APC protein is stabilized at the leading edge and controls microtubule organization by binding to their plus end. Consequently, the Golgi and MTOC relocate in front of the nucleus to establish the nuclear-centrosomal polarity axis typical of polarized migrating cells [27, 189]. Additionally, the polarity protein Dlg interacts with APC and promotes directed cell migration after being recruited by Cdc42-activated aPKC (Figure 6). The proposed mechanism is that Dlg mediates the binding between microtubule plus ends and the plasma membrane [40]. Notably, establishment of the nuclear-centrosomal polarity axis from the rear to the front is seen in cells utilizing both the mesenchymal and amoeboid invasion mode [158]. An exception are leukocytes that also migrate in an amoeboid manner, nonetheless their Golgi is located behind the nucleus [190], which points out the differences between amoeboid migration of immune cells and cancer cells.

In contrast to cell polarization where Cdc42 and Par complex play a central role, the role of polarity proteins in the regulation of Rho GTPases during cell migration seems to be more heterogeneous. In both epithelial and mesenchymal cells Par proteins directly regulate Rac activity by controlling its activator Tiam1. In epithelial cells, Rac1, Par3 and Tiam1 are needed for the formation of tight junctions [191]. Par3 was also proposed to recruit Tiam1 to the leading edge in migrating cells where it can activate Rac1 and initiate cell motility (Figure 6). The complex Par3-Tiam1 stabilizes the front-rear polarity in migrating cells and promotes directional migration [192]. The Par-Tiam signaling pathway is negatively regulated by Bcr, a Rac GAP, which interacts with both Rac and aPKC. Bcr regulates cell polarity by decreasing Rac activation and promoting degradation of aPKC at the leading edge. Not surprisingly, the loss of Bcr results in random polarized migration in astrocytes [193].

RhoA is also regulated, both positively and negatively, by polarity proteins in migrating cells. At the leading edge, RhoA is inhibited by Cdc42-activated Par6 and its downstream effector Smurf1. Par6 recruits Smurf1 to the leading edge which then locally degrades RhoA (Figures 6 and 7). Local degradation of RhoA increases the relative amount of RhoA at the cell rear [169, 194]. Reciprocally, RhoA inhibits the Par complex and Rac signaling. The RhoA effector ROCK phosphorylates Par3, thereby inducing disruption of the Par complex, deregulation of Rac activator Tiam1 and impaired Rac activation [195]. Polarity proteins are also involved in positive regulation of Rho. Par4 activates RhoA by interacting with its exchange factor Dbl, which results in actin filament assembly [196]. Moreover, Par4 also regulates Cdc42 as Par4 was shown to localize to the leading edge (Table 1) where it interacts with Cdc42 and maintains it in the active state [197].

### Other significant links between polarity complexes and cell migration

The Scribble complex promotes mesenchymal cell migration by increasing Rac1 and Cdc42 activity by the GEF βPIX [198] (Figure 6). In 3D collagen, βPIX also negatively controls RhoA activity through interaction with srGAP1 [199]. Scribble further participates in directional migration by targeting Cdc42 and Rac to the cell front. Cells with Scribble knockdown were unable to recruit Cdc42 and Rac to the leading edge causing impaired formation of lamellipodia and, in effect, impaired directional migration [200]. The function of Scribble is probably enhanced in cells that underwent EMT and express the mesenchymal marker vimentin. Scribble can bind to vimentin and this interaction protects Scribble from degradation [201]. The increased level of Scribble then supports directional migration. Paradoxically, membrane-localized Scribble can also suppress the invasive properties of Ras-induced human breast cells by inhibiting MAP/ERK signaling. Scribble that is not able to localize to the membrane and resides in the cytoplasm fails to suppress the MAPK/ERK signaling, E-cadherin expression and cell invasion [136].

Dlg, another member of the basolateral Scribble complex also relocates to the leading edge membrane during cell migration by interacting with PKCα. Both Dlg and PKCα were shown to be required for efficient polarized migration, although the molecular mechanism remains unknown [202]. Lgl also contributes to the regulation of front-rear polarity in migrating cells [203]. Lgl interacts with NMIIA (non-muscle myosin II A) in the lamella and also sequesters NMIIA from the leading edge of migrating cells. The sequestration of NMIIA from the leading edge prevents non-physiological assembly of NMIIA containing actin filaments and allows formation and maturation of focal adhesions, which enables efficient migration [203]. Interestingly, the interaction between Lgl and NMII is controlled by phosphorylation of Lgl by aPKC. Upon phosphorylation, Lgl dissociates from the Lgl1-Par6-aPKCζ complex at the leading edge and translocates to the lamella, where it interacts with NMIIA [204]. Of note, aPKCζ regulates organization of the actin cytoskeleton in migrating macrophages and leukocytes by activating LIMK and cofilin [205] (Figure 7). Whether this mechanism is shared by cancer cells utilizing the amoeboid invasion mode is not known.

### Planar cell polarity in single cell migration

Several studies have described the role of PCP proteins in tumor cell invasion. Both Vangl1 and Vangl2 associate with Scribble and at least Vangl2 participates in correct Scribble localization. Mutation in Vangl2 disrupts basolateral localization of Scribble, which results in impaired epithelium formation [206]. Vangl2 also interacts with Rac1 in epithelial cells, and the loss of either Rac1 or Vangl2 caused cell-cell adhesion defects [207]. Additionally, Vangl1 forms a ternary complex with nitric oxide synthase 1 adaptor protein (Nos1ap) and Scribble, and this complex localizes to lamellipodia at the leading edge (Figure 6). The Vangl1-Scribble-Nos1ap complex promotes directed invasion and the depletion of any of its components led to reduction of cell invasiveness [208].

Interestingly, there are reports indicating that Vangl2 controls endomembrane trafficking of several proteins involved in cell polarity and migration. Vangl2 promotes endocytosis of metalloproteinase MT1-MMP/MMP14 in fibrosarcoma HT1080 cells and during zebrafish development to control remodeling of the ECM [209]. Furthermore, Vangl2 suppresses protease-dependent collective cell invasion of cancer cells by reducing production of matrix proteases. Accordingly, loss of Vangl2 up-regulates the activity of secreted MMP2 [210]. In addition, Vangl2 enhances internalization of E-cadherin and N-cadherin [211]; however, the significance of this process remains unclear.

The localization of Vangl proteins in amoeboid invasion cells has not been thoroughly tested. However, in a cell line derived from B lymphocytes, which utilize the amoeboid migration mode, Vangl2 localized to the trailing edge [212] (Figure 7).

The activation of the non-canonical Wnt pathway by Wnt5 has a pleiotropic effect on cell invasion. During cell migration the Wnt5 receptor Frizzled accumulates at the leading edge of migrating cells where it interacts with integrins and binds the microtubule-associated protein APC through Dsh. Interestingly, the APC-Dsh complex was shown to associate with FAK and paxilin. Taken together, Wnt5-Fz regulate cellular adhesions through binding integrins and APC-Dsh [213]. Also, Wnt5a signaling contributes to organelle positioning and establishment of the migratory polarity during cell migration by phosphorylating Dsh, which leads to inactivation of GSK-3β. This signaling axis acts in synergy with the Cdc42-aPKC-mediated nucleus-MTOC-Golgi repositioning [47]. Furthermore, Wnt5 connects PCP to Rho GTPases and formins to promote cell invasion. Wnt5-activated Dsh further activates its downstream target, the formin Daam1, which can up-regulate the activity of RhoA [214] (Figure 7).

### Phosphoinositides in single cell migration

Phosphoinositides change their localization during the transition to the front-rear polarity (summarized in Table 1). In epithelial cells PTEN localizes to the apical membrane [215], while in migrating cells RhoA targets PTEN to the rear, where it degrades PIP3 and thus suppresses the activity of Rac1 and Cdc42 [216]. Reciprocally, Rho-ROCK was demonstrated to stimulate PTEN activity, which results in enhanced cell polarization [125]. Interestingly, Cdc42 is also able to induce PTEN localization to the front membrane, but in a much weaker manner, so it is detectable only after RhoA inhibition [125]. Furthermore, PTEN is known to affect cellular invasion by modulating the phosphorylation status of FAK [217], an important regulator of focal adhesions and mesenchymal invasion. The dephosphorylation of FAK by PTEN lead to altered cell-ECM adhesions and thus invasive behavior of migrating mesenchymal cells [217].

The action of PTEN could be overcome by PI3K. By producing PIP3 it can activate both Rac and Cdc42 [218]. PI3K associates with adherens junctions [219] in epithelial cells and translocates to the leading edge of motile cells. At the leading edge PI3K produces PIP3, and its accumulation results in Rac and Cdc42 activation. Integrin engagement to ECM within Rac-induced protrusions activates PI3K and PIP3 production, which subsequently further promotes Rac and Cdc42 activation in a positive feedback loop manner. Intriguingly, recent data suggest that in mesenchymally migrating cells, PI3K stabilizes already present cellular protrusions instead of initiating new ones. In this way, PI3K promotes persistent cell migration [220]. Consistently, Rac is necessary for accumulation of PIP3 at the cell front also in neutrophils, which migrate in an amoeboid manner [221]. The production of phospholipids further affects the localization of Par3. In epithelial cells, Par3 interacts with phospholipids to stabilize its localization to the TJ region [222], but whether phosphoinositides influence Par3 transfer to the leading edge in migrating cells has not yet been assessed.

### Polarity complexes and phosphoinositide signaling in collective cell migration

Similarly to individually invading cells, collective cell migration and invasion is also coupled with cell polarity signaling, although the role of polarity proteins in these processes is still incomplete. However, since following and leader cells in collectively migrating clusters display features typical of epithelial and mesenchymal cells, respectively, it is likely that identical or similar mechanisms that control epithelial or migratory polarity proteins are retained in collectively migrating cells. The cell cohort itself is polarized, as leader cells define the front, whereas increased actomyosin contractility is typically found at the back of the cell cluster. In the case where the cell cohort detaches from the epithelium, actomyosin contraction is induced at cell-cell contacts to generate adequate force for detachment of the cell sheet. This mechanism has been described in *Drosophila* development where Par1 promotes the detachment of border cells from epithelium by phosphorylating MLCP, which leads to enhanced actomyosin contraction that is needed for the initial detachment of the cell cluster [223]. Since the study was done in *Drosophila*, it would be of interest to find out whether the mechanism is also relevant for collective cancer cell invasion.

Once the cell cohort is moving, actomyosin contractility must be downregulated to maintain intercellular cohesion. DDR1 (discoidin receptor 1) was identified to be a major regulator of this process. DDR1 in complex with Par3/Par6 recruits RhoE to cell-cell contacts. RhoE antagonizes RhoA/ROCK signaling and thus actomyosin contractility. The depletion of DDR1 led to disrupted collective cancer cell invasion, which could be reversed by adding a ROCK inhibitor [224]. However, DDR1 can also promote single cell migration by up-regulating N-cadherin [225], suggesting that its role in cancer cell migration could be context or cell type dependent.

Furthermore, tight junction protein occludin was shown to influence directional migration of cell sheets during a wound healing assay. Occludin activates PI3K at leading edge by localizing Par3 and aPKC through PATJ to the cell front [226].

## CLINICAL SIGNIFICANCE OF POLARITY COMPLEXES IN MALIGNANT INVASION AND METASTASIS

Given the overall importance of polarity proteins for migration, it is not surprising that their altered function can be correlated with the development of aggressive metastatic disease with poor prognosis. For example, the down-regulation of Par3 in breast cancer cell lines showed that the invasiveness of ErbB2-positive cells was higher in cells lacking Par3 expression [227]. The decreased Par3 level was also found to result in reduced latency of tumorigenesis in murine mammary gland cells with activated Ras or Notch pathways. The elevated tumor potential and invasive phenotype was caused by delocalization of aPKC and its activation of Stat3, which was accompanied by elevated MMP-9 [228]. Compromised Par3 function has also been associated with increased invasive and metastatic potential of squamous cell lung carcinomas, although in this case Stat3 activity was reduced [229]. Similarly, attenuating Par complex by SHP2 phosphatase resulted in EMT and promoted metastasis formation of prostate cancer cells [230]. These studies indicated that Par3 could act as a metastasis suppressor. However, the Par3 function seems to be cancer type specific since Par3 over-expression in kidney and liver tumors correlated with poor patient outcome [231, 232].

It is of interest that not only Par3 is associated with elevated MMP levels. Par6 promotes aPKC activity, which was correlated with the levels of MMP-10 in non-small cell lung cancer. Blocking the aPKC kinase function diminished the MMP-10 levels [233]. Analogously, in triple negative breast cancer cell lines aPKC controlled the level of MT1-MMP by enhancing vesicular trafficking of the metalloprotease. In cells with silenced aPKC, a two-fold drop of the amount of MT1-MMP-positive endosomes was observed. Importantly, the upregulation of both MT1-MMP and aPKCι inversely correlate with metastasis-free patient survival [234]. Moreover, aPKCζ mediates the recruitment and activation of MMP-9 and MMP-14 to the sites of podosomes [235], which supports proteolytic dependent cell invasion (Figure 4). In human lung adenocarcinoma, aPKC co-localizes to the apical membrane along with Lgl. The apical localization of Lgl was correlated with increased lymph node metastasis [236]. On the other hand, in colorectal carcinoma higher incidence of lymph node metastasis was observed in tumors with loss of Lgl [237].

The loss of Par4 has been shown to promote not only cancer growth, but also the initial loss of polarity. It was demonstrated that Par4 loss leads to mislocalization of serine protease Hepsin, which resulted in disrupted integrity of the basement membrane [238]. Consistently, the downregulation of Par4 in breast cancer resulted in an invasive phenotype with impaired polarity [239]. Another study showed that Par4 interacts with p114RhoGEF to control RhoA activity during the formation of apical junctions. The deprivation of Par4 leads to the loss of epithelial integrity by disrupting assembly of apical adhesions [240]. Accordingly, overexpression of Par4 inhibits invasion and reduces tumor growth of breast cancer cells by reducing the levels of MMP-2 and MMP-9 [241].

The PCP component Vangl1 has also been shown to promote cell migration and invasion in murine models of colorectal and squamous cell carcinoma [242, 243].

## TARGETING POLARITY COMPONENTS IN CANCER

The crucial role of cancer cell invasion in metastatic disease makes this process a valuable target for therapeutical intervention. Indeed, development of anti-metastatic therapeutics includes targets such as polarity complexes, EMT or Rho GTPases. At present, drugs targeting aPKC are under development (reviewed in [183]). One of them is aurothiomalate, which blocks the interaction between PKCι and Par6 [244]. Aurothiomalate has successfully passed phase I trials [245].

EMT is a promising target for new therapeutic interventions because it plays an important, if not central, role at several steps of the metastatic cascade (Figure 1). In addition to invasiveness, EMT has also been implicated in survival of circulating tumor cells or survival of cancer cells after ionizing radiation [246, 247]. Accordingly, agents targeting the TGFβ pathway or transcription factors Snail/Slug, Twist or cadherins are being tested [248–251]. An interesting complementary approach is not to block EMT and tumor dissemination, but promote MET instead. This was recently shown to be effective in melanoma [252].

Drugs against Rho GTPases were designed to target their prenylation, modify their activation by recruiting inhibitory proteins GDIs or GAPs, or less often by direct interaction with the particular Rho GTPase [253]. None of the tested drugs has reached clinical trials yet. However, recently, an allosteric inhibitor of Rac and Cdc42 R-ketorolac was shown to reduce ovarian cancer cell invasion *in vitro* [254].

The necessity of proteolytic activity for mesenchymal migration was investigated in the context of anti-cancer drugs. Several identified inhibitors of MMPs blocked mesenchymal migration [255]. However, they turned out to be largely ineffective in clinical trials, partially because of problematic bioavailability, side effects and administration in advanced stages, but also due to the evaluation criteria. Scoring the effect of inhibitors according to tumor shrinkage often omits the effect on much more important and relevant aspects of the disease - tumor invasion and metastasis [256, 257]. Nevertheless, blocking MMPs in cell lines *in vitro* led to the identification of the cells ability to shut down the mesenchymal mode of migration and utilize the amoeboid mode [99].

An additional clinical challenge is the prediction of tumor progression. One prognosis factor is E-cadherin, which is lost during the progression to metastatic disease. Further prognostic factors are Crumbs3 and Par3, whose altered expressions have been correlated with the level of metastasis [232, 258]. Also, increased expression of a pro-invasive isoform of the actin binding protein MenaINV has been correlated with the gain of metastatic characteristics by inducing trans-endothelial migration [259].

## CONCLUDING REMARKS

Loss of epithelial polarity and de-differentiation of epithelial cells are crucial for establishment of the migratory and invasive polarity, which underlies efficient metastatic spread of cancer cells. Evidence suggests that cell polarity proteins, Rho GTPases, phosphoinositides and their associated signaling networks cooperate to establish the front-rear polarity and promote cancer cell invasion (Figures 6 and 7). Apparently, specific localization of polarity proteins within the cell, their mutation, silencing or overexpression is decisive for the cell fate and can either help maintain the epithelial program or support the transition towards an invasive pathological phenotype (Table 1).

Cancer is generally viewed as a result of genetic and epigenetic changes that activate oncogenes and inactivate tumor suppressors. Over the years it has also become evident that signals from the tumor microenvironment, represented by associated cells, diverse biochemical signaling and the extracellular matrix, are important factors that promote or oppose tumorigenesis by cooperating with or dominating over both genetic and epigenetic alterations of cancer cells [260]. This is particularly evident in the case of cell invasion, where the physical and biochemical characteristics of the ECM are able to dictate the tumor cell invasion strategy. All the more so, it is necessary to study cells in complex 3D environments, since the localization of proteins can be affected by experimental conditions [261]. Indeed, the ECM organization challenges cells with different topological surfaces, and tumor cells adopt different polarized invasion phenotypes when migrating on narrow single fibers (1D environment), on planar substrates (2D), or within the fibrillary meshwork (3D). This topic was not extensively discussed here and it has been covered recently in several excellent reviews [85, 262–264]

The emerging picture is that the preferred invasion mode adopted by the cancer cell is the net result of extracellular cues, which decipher the physical properties of ECM, along with intracellular signaling featuring the cell polarity signaling components as the main players. The challenge now is to learn how these physical and chemical signals along with the polarity and migration machinery are mutually coordinated and integrated to control the so diverse, yet so similar modes of tumor cell invasion.
